# Insights into bacterial stress adaptation, host interactions, and drug resistance: key findings from the fall 2025 ASM Theobald Smith Society meeting

**DOI:** 10.1128/msphere.00878-25

**Published:** 2026-03-06

**Authors:** Nitish Sharma, Madhulika Singh, Joycelyn Radeny, Arpita Mukherjee, Raymond F. Sullivan, Jeffrey M. Boyd, Valerie J. Carabetta, Jason H. Yang, Srujana S. Yadavalli

**Affiliations:** 1 Waksman Institute of Microbiology, Rutgers University 57675, Piscataway, New Jersey, USA; 2 Department of Microbiology, Biochemistry and Molecular Genetics, New Jersey Medical School, Rutgers University 67206 , Newark, New Jersey, USA; 3 Department of Marine and Coastal Sciences, Rutgers University 5970, New Brunswick, New Jersey, USA; 4 Department of Biochemistry and Microbiology, Rutgers University 5970, New Brunswick, New Jersey, USA; 5 Department of Biomedical Sciences, Cooper Medical School of Rowan University 363994, Camden, New Jersey, USA; 6 Center for Emerging Pathogens, New Jersey Medical School, Rutgers University, Newark, New Jersey, USA; 7 Department of Genetics, Rutgers University 198473, Piscataway, New Jersey, USA; Virginia-Maryland College of Veterinary Medicine, Blacksburg, Virginia, USA

**Keywords:** branch meeting, microbiology, molecular biology, cell biology, microbial physiology, microbiome, applied microbiology, antimicrobial resistance

## Abstract

The annual fall meeting for the Theobald Smith Society (TSS), the New Jersey Branch of the American Society for Microbiology (ASM), took place in November 2025 at Cooper Medical School of Rowan University in Camden, New Jersey. A total of 72 branch members from across New Jersey participated, including undergraduate and graduate students, postdoctoral trainees, faculty, and professionals from government and industry. This report highlights the scope and diversity of research carried out by TSS members and celebrates their impactful discoveries.

## INTRODUCTION

## MEETING SUMMARY INFORMATION

The annual fall meeting for the Theobald Smith Society (TSS), the New Jersey Branch of the American Society of Microbiology (ASM), was held in November 2025 and was hosted for the first time by Cooper Medical School of Rowan University (CMSRU) in Camden, NJ. TSS comprises members across New Jersey. This year, there were 72 attendees, with 40 undergraduate and graduate students, 11 post-doctoral trainees, 15 faculty, and 6 staff, visiting and/or industry members (Table 1). Attendees represented various institutions across New Jersey, including Rutgers University-New Brunswick, Rutgers University-Newark, Rutgers University-Camden, Rutgers University-New Jersey Medical School, Montclair State University, Cooper Medical School of Rowan University, Princeton University, and Hackensack Meridian Health (Table 2). The meeting schedule for the Fall 2025 TSS symposium is presented in Table 3. The morning keynote talk, entitled “Chewing the fat: lipids as second messengers in *Staphylococcus aureus* signal transduction,” was presented by Dr. Shaun Brinsmade, an associate professor of biology at Georgetown University and an ASM distinguished speaker. This was followed by 10-min invited trainee talks by Dr. Nadine Alvarez (Hackensack Meridian Health), Lylla Almosd (Rutgers University-New Brunswick), and Dr. Rabindra Khadka (Rutgers University-New Brunswick). The afternoon keynote talk, titled “Metabolic vulnerabilities in drug-resistant tuberculosis,” was presented by 2025 TSS Young Investigator awardee Dr. Jason Yang, an assistant professor and Chancellor Scholar of microbiology, biochemistry and molecular genetics at Rutgers University-New Jersey Medical School. The 10-min invited trainee afternoon talks were delivered by Madhulika Singh and Arpita Mukherjee (Rutgers University-New Brunswick), DiemQuynh Nguyen (Rutgers University-Newark), and Bala Madduri (Rutgers University-New Jersey Medical School). Afternoon talks were followed by 45 poster presentations from various institutions across New Jersey. The meeting concluded with the announcement of poster award winners (Table 4) and closing remarks, thank yous to organizers, and the announcement of Dr. Jason Yang as the new TSS president-elect.

**TABLE 1:** Career stage of symposium attendees

Career stage	Count	%
Graduate student	29	40
Faculty	15	21
Post-doctoral trainee	11	15
Undergraduate student	10	14
Visiting scientist	2	3
Industry	2	3
Lab technician	1	1
MD/PhD student	1	1
Staff	1	1
**Total**	**72**	

**TABLE 2:** Affiliations of symposium attendees

Affiliation	Count	%
Rutgers University-New Brunswick	25	35
Montclair State University	12	17
Rutgers-New Jersey Medical School	6	8
Rutgers University-Camden	6	8
Rowan University	5	7
Rutgers University-Newark	5	7
Hackensack Meridian Health	3	4
Cooper Medical School of Rowan University	2	3
Princeton University	2	3
Industry	2	3
New York Institute of Technology	1	1
Rowan School of Osteopathic Medicine	1	1
Rutgers University-Robert Wood Johnson Medical School	1	1
Seton Hall University	1	1
**Total**	**72**	

**TABLE 3:** Fall 2025 TSS meeting schedule

Time	Scheduled item
9:45 AM	Registration begins
10:15 AM	Opening remarks by TSS President Srujana S. Yadavalli
10:30 AM	ASM Distinguished Lecture by Shaun Brinsmade
11:30 AM	Invited speaker: Nadine Alvarez
11:45 AM	Invited speaker: Lylla Almosd
12:00 PM	Invited speaker: Rabindra Khadka
12:15 PM	Lunch and networking
1:15 PM	Young Investigator Award Lecture by Jason H. Yang
2:00 PM	Invited speaker(s): Madhulika Singh and Arpita Mukherjee
2:15 PM	Invited speaker: DiemQuynh Nguyen
2:30 PM	Invited speaker: Bala Madduri
2:45 PM	Poster session I (odd numbers)
3:30 PM	Poster session II (even numbers)
4:15 PM	Poster award announcements and closing remarks

**TABLE 4:** List of poster prize winners

Name	Trainee stage	Affiliation
Alexandra Everson	Graduate (master’s)	Montclair State University
Carlos Resstel	Graduate (Ph.D.)	Rutgers-New Jersey Medical School
Brynn Sillick	Undergraduate	Rutgers University–New Brunswick
Chioma Uchendu	Graduate (Ph.D.)	Rutgers University–Camden

## MEETING ATTENDANCE HISTORY

**Fig 1:**
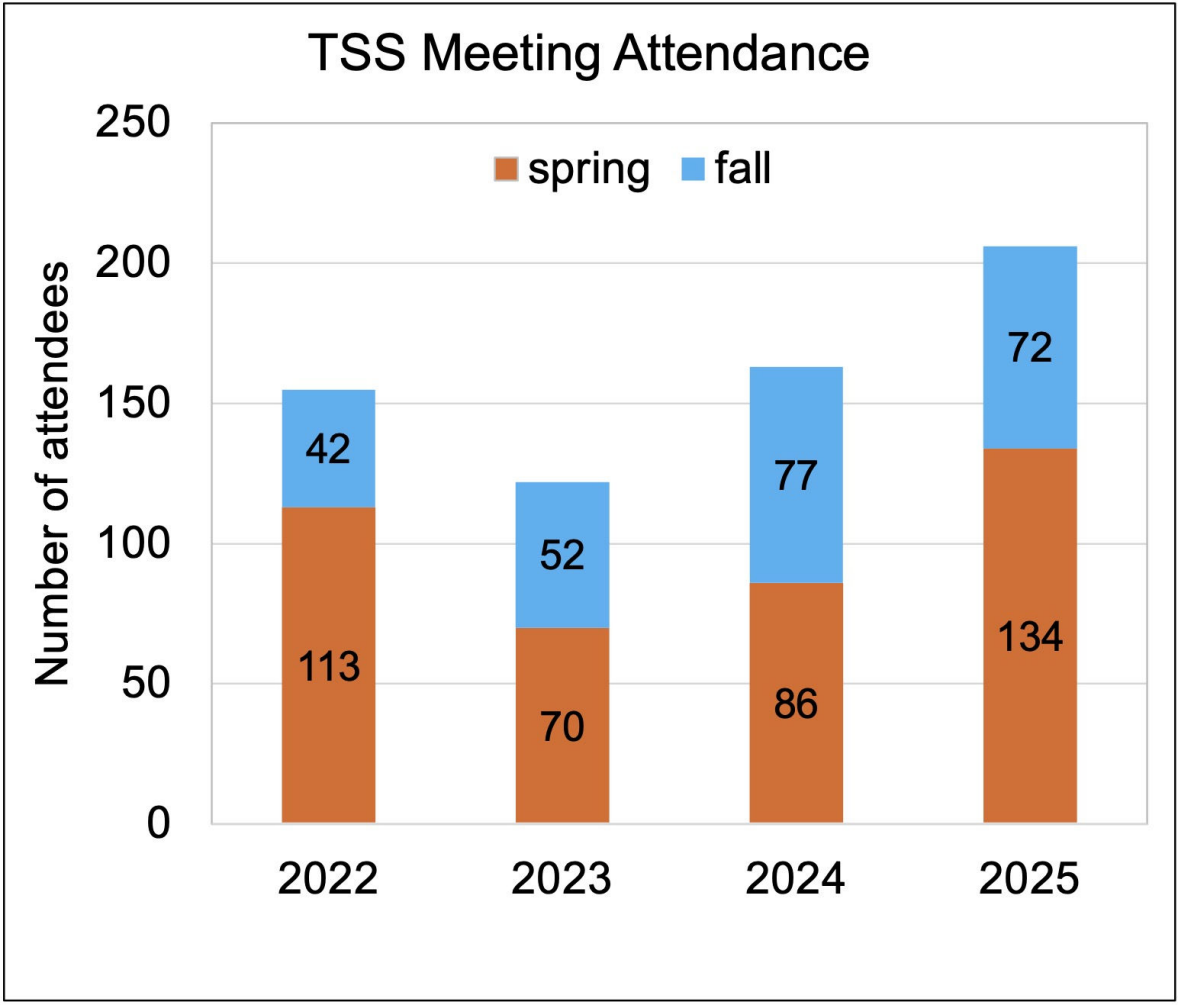
Theobald Smith Society (TSS), the NJ Branch of ASM, meeting attendance. The number of attendees at the spring and fall meetings of TSS is indicated for the past 4 years.

Meeting attendance has been stable historically, with significant growth in recent years (Fig. 1). Spring meetings are typically held at Rutgers University–New Brunswick due to its location central in New Jersey, while fall meetings rotate among institutions across New Jersey to engage the broader TSS community and accommodate attendees from diverse institutions. Venue accessibility and location influence attendance patterns, with centrally located sites attracting larger participation. In spring 2025, we partnered with the Rutgers-wide Microbiology Symposium to hold back-to-back events, effectively doubling attendance compared to the previous year at the same venue (Fig. 1). This growth, alongside increased poster presentations, reflects sustained engagement and our Society’s expanding geographic and topical reach.

## OPENING REMARKS

As the host and treasurer of the TSS, Dr. Valerie Carabetta, associate professor of biomedical sciences at CMSRU, opened the meeting with a brief introduction to CMSRU. She highlighted the medical and research programs they offered and added that CMSRU is a “leader in innovation for medical education and biomedical research” with a mission deeply connected to community engagement.

As the 82nd president of the TSS, Dr. Srujana Yadavalli, assistant professor in the Rutgers University Waksman Institute of Microbiology, welcomed attendees to the symposium and thanked the organizing committee. She introduced TSS, named after pioneering bacteriologist Theobald Smith, whose 280+ publications established the field of host-pathogen interactions in the United States. She reflected on the successful spring 2025 meeting, highlighting strong student and postdoc participation, including poster prize winners and six trainee lightning talks.

Dr. Yadavalli encouraged attendees to explore ASM membership for professional development, leadership opportunities, and career support. She announced that the 2026 ASM Microbe meeting would be held in Washington, DC, with abstract submissions opening mid-November, and described the Peggy Cotter Travel Awards—branch grants helping early-career microbiologists attend national meetings.

She introduced two ASM journal representatives, Adriana Borgia and Joe Schwartz, who would answer publication-related questions, and announced two new ASM journals: *ASM Animal Microbiology*, focused on microbes influencing animal and public health, and *ASM Food Microbiology*, covering foodborne pathogens, food microbiomes, and safety research.

Dr. Yadavalli previewed the day’s program, featuring six short talks and posters by trainees and keynote speakers: ASM Distinguished Lecturer Dr. Shaun Brinsmade of Georgetown University in the morning and 2025 Young Investigator Award recipient Dr. Jason Yang in the afternoon. She then formally introduced Dr. Brinsmade, summarizing his distinguished career studying regulatory networks and metabolism in *Staphylococcus aureus* and his contributions as a mentor and scholar.

## KEYNOTE ADDRESS 1: ASM DISTINGUISHED LECTURE

Dr. Shaun Brinsmade, Ph.D., Provost’s Distinguished Associate Professor at Georgetown University (Department of Biology), delivered the ASM Distinguished Lecture, entitled “Chewing the fat: lipids as second messengers in *Staphylococcus aureus* signal transduction”, focusing on understanding how *S. aureus* utilizes nutrient sensing and membrane-derived lipid signals to regulate virulence. His research highlights how the transcription factor CodY integrates metabolic cues, particularly branched-chain amino acids (isoleucine, leucine, and valine) and their cognate branched-chain fatty acids, to modulate the SaePQRS two-component system, a critical regulator of toxin production and neutrophil evasion (1).

Dr. Brinsmade detailed the substantial global burden of *S. aureus*, which contributes to over 700,000 deaths annually and is responsible for three-quarters of skin and soft tissue infections worldwide. As a commensal colonizer of the posterior nares with extensive antimicrobial resistance, including methicillin-resistant *S. aureus* (MRSA) strains capable of human-animal transmission, *S. aureus* poses a significant public health concern. It can colonize virtually every surface of the body because of a wide array of surface-associated and secreted factors, and it adapts to different host niches by secreting toxins, proteases (which liberate host nutrients), adhesins, autolysins, leucocidins, and immunoglobulin-binding proteins. This adaptability, together with its resistance to multiple antimicrobials, imposes a significant financial burden on healthcare systems, making *S. aureus* a valuable model for understanding how pathogens adapt to diverse infection niches. The timing of virulence factor production is tightly regulated by small regulatory RNAs (sRNAs), transcription factors, and two-component systems (TCS) (2). Central to the talk was CodY, a Firmicutes-specific transcription factor that functions as a nutrient sensor and transcriptional regulator of both metabolism and virulence genes. CodY binds GTP and the branched-chain amino acids (BCFAs)—isoleucine (I), leucine (L), and valine (V)—to monitor intracellular nutrient and energy status. ILV are abundant, hydrophobic amino acids that contribute to protein folding and feed into metabolic pathways that generate fatty acids, proteins, pantothenate, and coenzyme A; by binding ILV, CodY gages nutrient sufficiency. *S. aureus* exhibits pseudo-auxotrophy for ILV: although it encodes the biosynthetic genes for ILV, it relies on external ILV (at least under laboratory conditions) for cellular processes. During infection, ILV (particularly isoleucine) may serve as a signal to inform its location and metabolic state, and thereby tune virulence programs. CodY restrains production of potent virulence factors indirectly by repressing the SaePQRS two-component system, which defends *S. aureus* against neutrophil-secreted peptides and proteins and controls numerous virulence determinants. The Sae system consists of the intramembrane histidine kinase SaeS and the response regulator SaeR, with SaeP and SaeQ acting as negative regulators. CodY represses the *sae* locus in part by inhibiting the P1 promoter upstream of *sae*; the P3 promoter near saeQ is relatively constitutive and contributes to basal Sae expression (3). Functional assays demonstrate that culture filtrates from wild-type *S. aureus* kill neutrophils, whereas deletion of *saeR* or combined deletion of *codY* and *saeR* reduces neutrophil death; deletion of codY alone does not reduce killing relative to wild type, indicating that SaeRS activity is essential for the neutrophil-killing phenotype and that CodY’s effect on virulence is mediated through the SaeRS system.

A key mechanistic question addressed by Dr. Brinsmade’s group was whether membrane lipids act as second messengers to regulate Sae signaling. Because SaeS is an intramembrane kinase, membrane composition is a plausible regulator of its activity. The team focused on BCFAs, which form the hydrophobic tails of membrane lipids in *S. aureus*, fluidize the membrane, and are required for successful single-species skin and soft tissue infections. Using phos-tag electrophoresis, the group showed elevated SaeS phosphorylation (and thus increased kinase activity) in *codY* deletion strains, indicating that CodY normally restrains Sae signaling. Gas chromatography for fatty acid methyl esters analysis revealed altered BCFA composition in ∆*codY* and in mutants defective for BCFA synthesis (for example, ∆*lpdA mbcS1*), with loss of CodY associated with a significant increase in BCFA content. Sae kinase activity positively correlates with BCFA branching, and the effect is specific to particular BCFAs (for example, 15:0 anteiso-species). These data support a model in which CodY controls ILV metabolism and precursor flux into BCFA synthesis, and these membrane lipids, in turn, modulate SaeS kinase activity. Genetic and biochemical perturbations further support this CodY-ILV-BCFA-Sae axis. Blocking *de novo* ILV biosynthesis and restricting isoleucine transport reduces Sae TCS activity as measured by P*_nuc_*-GFP reporter assays; conversely, overexpression of the isoleucine transporter BrnQ2 increases Sae activity. Loss of CodY increases BCFA levels, and CodY requires BCFAs to upregulate SaeS kinase activity. When BCFA precursor production is inhibited, Sae activity is reduced, indicating that membrane lipid composition acts as an integrated signal that determines when *S. aureus* unleashes virulence programs through the Sae two-component system. The novel aspects of these findings are that they identify BCFAs as second-messenger lipids that influence a membrane-embedded sensor kinase, and they reveal how a nutrient-sensing transcription factor (CodY) controls membrane lipid composition to regulate virulence. These insights explain how *S. aureus* uses nutrient sensing to restrain or unleash pathogenic potential *via* Sae and highlight the SaeRS TCS as an attractive anti-virulence target. Anti-virulence strategies, such as inhibitors of SaeRS or BCFA synthesis, are gaining traction because they exert less selective pressure, are selective, exhibit synergy with conventional antibiotics, and cause less disruption of the microbiota. For example, targeting SaeRS can inhibit the production of clinically important toxins, such as toxic shock syndrome toxin-1. Therapeutically, disrupting the CodY-BCFA-Sae axis could sensitize *S. aureus* to membrane-targeting antimicrobials while minimizing collateral damage to beneficial microbes. Ongoing work in the Brinsmade lab investigates the interactions between Sae components and membrane scaffold proteins, such as FloA, to gain a deeper understanding of the spatial regulation of signal transduction.

## KEYNOTE ADDRESS 2: 2025 YOUNG INVESTIGATOR AWARD LECTURE

Dr. Jason Yang, assistant professor and Chancellor Scholar of Microbiology, Biochemistry & Molecular Genetics at Rutgers University-New Jersey Medical School, gave the Young Investigator Award lecture, entitled “Metabolic vulnerabilities in drug-resistant tuberculosis.” Tuberculosis (TB), caused by the bacterial pathogen *Mycobacterium tuberculosis* (Mtb), is the leading cause of death by a single infectious agent, resulting in 10.7 million infections and 1.2 million deaths annually (4). Despite the availability of effective first-line antibiotics (rifampicin, isoniazid, pyrazinamide, ethambutol), drug resistance is increasing, with 390,000 annual rifampin-resistant and multidrug-resistant (MDR-TB) cases. One recently discovered antibiotic was recently approved by the World Health Organization for treating MDR-TB: the diarylquinoline bedaquiline, which inhibits mycobacterial ATP synthase. Interestingly, MDR-Mtb clinical strains are hyper-sensitive to bedaquiline when compared with drug-susceptible strains. Dr. Yang’s team investigated the mechanisms underlying this hypersensitivity using systems biology approaches.

During infection, Mtb expresses catalase-peroxidase (encoded by *katG*) to detoxify host-induced oxidative stress. KatG is also needed to activate the first-line TB antibiotic isoniazid, which inhibits Mtb cell wall synthesis. Approximately 70% of isoniazid-resistant cases involve katG mutations. Dr. Yang’s team developed a *katG* deletion mutant to model isoniazid resistance (5). By analyzing drug susceptibility measurements from over 12,000 Mtb clinical isolates, they found that isoniazid resistance was associated with increased bedaquiline susceptibility. The *katG* deletion mutant showed increased bedaquiline sensitivity compared to wild-type, suggesting that catalase deficiency was sufficient for causing the bedaquiline hyper-sensitivity in MDR-Mtb. Complementation of *katG* restored catalase activity and restored bedaquiline drug tolerance, establishing causality. They observed increased reactive oxygen species (ROS) and oxidative cellular damage in the *katG* deletion mutant.

RNA sequencing revealed that *katG* deletion altered the expression of transcription factors before drug exposure, including regulators like Rv3160c. They found that overexpression of Rv3160c enhanced bedaquiline sensitivity, suggesting that catalase activity regulates transcriptional programs that can also regulate drug susceptibility. Catalase-deficient strains also showed baseline suppression of DNA repair genes that were more strongly induced following drug exposure relative to wild-type cells. They found that bedaquiline treatment induced oxidative stress response genes, elevated ROS formation, and increased protein carbonylation and DNA oxidation. Inhibiting ATP synthesis unexpectedly increases redox stress because energy-starved cells push oxidative phosphorylation harder, amplifying ROS production by NADH dehydrogenase. This cascade—loss of catalase activity compromising redox balance and DNA repair—makes cells vulnerable to energetic stress from ATP synthase inhibition.

By applying constraint-based genome-scale metabolic modeling techniques to their transcriptomic data, they predicted that folate biosynthesis would be disrupted in catalase-deficient strains at baseline and would be further impaired under ATP synthase inhibition. Validation experiments confirmed hypersensitivity to folate-pathway inhibitors (trimethoprim, sulfonamides) in catalase-deficient mutants. Notably, this hypersensitivity disappeared in fully MDR strains, suggesting that secondary mutations in RNA polymerase (associated with rifampicin resistance) compensate for these folate biosynthesis defects by remodeling Mtb metabolism.

Dr. Yang highlighted a Norwegian longitudinal study tracking Mtb genomic and transcriptomic evolution in one patient over 4 years. In an unpublished analysis, their metabolic modeling analyses revealed progressive remodeling of cell wall, redox metabolism, and folate biosynthesis pathways as drug-susceptible Mtb became multidrug-resistant and pre-extensively drug-resistant, reinforcing the premise that drug resistance reshapes bacterial metabolism and can create druggable vulnerabilities.

Dr. Yang concluded that systems biology approaches can identify metabolic weak points in resistant pathogens. Their analyses suggest that pathways like menaquinone biosynthesis and energy cofactor metabolism represent promising drug targets. By integrating experimental activities with computational modeling, they aim to better understand the bacterial physiology of drug-resistant pathogens and propose that these analyses can uncover hidden liabilities that can become exciting new drug targets.

## PRESENTATION THEMES

The oral and poster presentations were well-distributed across the three scientific units defined by ASM, reflecting the breadth of research represented at the meeting. Among 38 poster presentations, the largest share (28 posters) fell under Microbial Mechanism Discovery, underscoring the community’s continuing emphasis on fundamental insights into microbial physiology, genetics, and host–microbe interactions, while Health Microbiology (22 posters) and Applied and Environmental Microbiology (12 posters) were also strongly represented. Substantial thematic overlap demonstrated how research today increasingly bridges traditional boundaries: 18 posters connected mechanistic discovery with health relevance, 4 linked mechanisms with environmental or applied topics, 3 combined health and environmental perspectives, and, notably, 1 poster integrated all three domains. Overall, this distribution reveals a vibrant mix of fundamental, translational, and applied microbiology that mirrors the ASM’s core focus areas and emphasizes how investigators are drawing connections between molecular mechanisms, clinical outcomes, and environmental impact.

## ASM MECHANISM DISCOVERY

Mechanism Discovery featured most of the talks and poster presentations. It covered diverse topics, such as cellular physiology, molecular genetics, protein structure-function studies, metabolism, systems biology, and microbial evolution.

Bala Madduri (Rutgers University-Newark) presented her work on the role of *M. tuberculosis* secreted effector protein PE5 in modulating macrophage endosomal recycling via CRL2 ubiquitin machinery. *M. tuberculosis* prevents phagocyte killing and takes over macrophage biology via PE/PPE effector proteins. The PE/PPE effector proteins are crucial for pathogenesis, conserved across several mycobacterial species (*M. tuberculosis*, *M. bovis, M. marinum,* and *M. leprae*), and occupy 10% of the genome. However, these effector proteins are largely uncharacterized. Molecular function of PE5, a member of the PE/PPE family, was elucidated in this work. PE5 interacts with the host CRL2-KLHDC2 ubiquitin ligase complex through C-terminal diglycine. Intracellular replication of *M. tuberculosis* within macrophages is dependent on the CRL2-KLHDC2 complex. PE5 undergoes proteasomal degradation independent of canonical ubiquitination, by dragging a non-CRL2 substrate for degradation. Future studies will focus on identifying the specific host proteins destabilized by PE5 and determining whether other PE/PPE family members use similar strategies to manipulate host ubiquitin systems.

Rabindra Khadka (Rutgers University-New Brunswick) shared mechanistic insights on general stress response in *Bacillus subtilis*, presenting findings that challenge long-held assumptions about the established stress response model. During non-lethal environmental stresses, cytoplasmic complexes called stressosomes induce Sigma B-mediated general stress response in *B. subtilis*. Stressosome protein RsbR has been linked with stress sensing and phosphorylation of RsbS via RsbT kinase. Subsequently, RsbT is released from the stressosome complex and activates σB. This study shows that the substitutions in the N-terminal of RsbR (the primary sensor paralog within the stressosome) affect the magnitude and duration of the σB activity in response to ethanol stress. However, cells lacking *rsbR* paralogs and *rsbS* still display robust σB activity during ethanol stress, suggesting that the stressosome is not essential for sensing stress itself but rather serves as a modulator that fine-tunes the timing and magnitude of σB activation. When RsbT itself was deleted, the stress response was completely abolished. Complementing the mutant with an inducible copy of RsbT restored normal stress sensing, confirming that RsbT is necessary and sufficient for environmental stress detection and mediates stressosome-independent activation of σB in response to stress. Single-cell microfluidic microscopy revealed that cells lacking the stressosome displayed pulsatile σB activation, resulting from the feedback loops between σB, its phosphatase, and kinase regulators, independent of stressosome mediation. In fact, in the revised model, RsbT alone can sense stress and modulate σB response. Further studies are required to establish the mechanism of stress sensing by RsbT. Homologous RsbT elements appear in other taxa, including *Staphylococcus* and *Vibrio*, where they play roles in oxygen sensing, suggesting broader implications for understanding bacterial stress adaptation.

Dr. Madhulika Singh and Arpita Mukherjee (Rutgers University-New Brunswick) presented their joint research on probing the mechanism of stress-induced filamentation in *Escherichia coli*, focusing on the PhoQ/PhoP two-component signaling system, which regulates magnesium homeostasis, acid tolerance, and resistance to cationic antimicrobial peptides. They discussed that filamentation, a bacterial survival strategy, can be stimulated in the presence of antimicrobial peptides and is mediated by QueE. QueE, known for its role in queuosine (Q) tRNA modification, moonlights as a cell division inhibitor. It co-localizes with the divisome protein FtsZ and affects the recruitment of late-stage divisome components, including FtsQ, FtsW, and FtsI. Fluorescence imaging revealed irregular localization patterns of the mid-stage divisome protein FtsK during QueE overexpression, indicating that QueE interferes with FtsK’s recruitment or stability. FtsK is known to interact with an outer membrane lipoprotein RlpA, and deletion of RlpA partially suppresses the filamentation phenotype, suggesting that QueE disrupts the FtsK-RlpA interactions. The elongated cells remain viable and revert to normal size once stress is removed. They show that filamented cells display differential tolerance to antibiotics targeting late stages of cell division. Ongoing experiments involve bacterial two-hybrid assays and protein localization studies to confirm physical interactions among QueE, FtsK, and RlpA. Their work sets the stage for future studies on how PhoP/PhoQ activation modulates divisome organization during stress-induced filamentation.

Chioma G. Uchendu (Rutgers University-Camden) was awarded a poster prize for her work on the spatial organization of sphingolipid synthesis in bacteria. Her study sought to determine the steps in bacterial sphingolipid biosynthesis by examining the localization of three core enzymes. Through bioinformatic analysis of sphingolipid biosynthesis genes, integrating lipid profiling and structural modeling, along with subcellular localization assays using β-lactamase fusions and permeabilization experiments, she was able to map out where each enzyme is located within the cell. The work reveals that serine palmitoyl transferase and ceramide synthase reside in the cytoplasm, while ceramide reductase localizes to the periplasm. This spatial separation supports a model in which acyl chain addition precedes long-chain base reduction, thereby indicating a pathway that is distinct from eukaryotic sphingolipid biosynthesis. These data additionally suggest that the second acyl chain is attached in the cytoplasm before the intermediate translocates for final reduction in the periplasm, adding to the depth of the directional flow for this distinctive bacterial lipid pathway.

Brynn Silick (Rutgers University-New Brunswick) was awarded a poster prize for her work on a broadly applicable strategy to investigate how hybrid two-component systems (HTCSs) are able to regulate glycan sensing in *Bacteroides* species. By generating constitutively active HTCS variants and utilizing bioinformatic modeling with experimental mapping of DNA-binding profiles and regulatory networks, she was able to characterize two representative HTCSs, BT4124 and BT1734. Her work reveals glycan-utilization pathways that are beyond classical polysaccharide utilization loci. Furthermore, her work developed a framework that provides a universal approach to decode HTCS signaling networks and understand how *Bacteroides* species coordinate complex glycan metabolism.

Carlos Resstel (Rutgers University-New Jersey Medical School) was awarded a poster prize for his work on PE9, which was identified as a nuclear effector protein in *M. tuberculosis* from a screen of 12 PE/PPE proteins predicted to contain nuclear localization signals—only PE9 localized to the host nucleus. PE9 contains a bipartite nuclear localization signal (a single signal with two basic regions) and interacts with nuclear transport proteins that are both required for nuclear localization. PE9 alters IFN-β expression via interaction with cyclic GMP-AMP synthase (cGAS), revealing a novel immune evasion mechanism in tuberculosis infection. His work elucidates the role of PE9 in host immune modulation.

Alexandra Everson (Montclair State University) was awarded a poster prize for her work on the effects of E47D and E157D mutants in IGPS (indole-3-glycerol phosphate synthase) catalytic activity from hyperthermophilic bacteria, *Thermotoga maritima*. Her study focused on analyzing the effects of single-point mutations to identify important binding residues in IGPS that catalyze the essential tryptophan biosynthesis pathway. Mutants E47D and E157D had reduced catalytic activity compared with wild type under normal conditions. E47D mutation resulted in a 2.3-fold increase in activity at higher glycerol concentrations and a fivefold increase in activity when the reaction is performed in D2O at 55°C. IGPS is conserved in pathogens, such as *M. tuberculosis,* where it is important for the bacterium’s survival and growth. Such studies can potentially identify targets for inhibitor design against diseases such as tuberculosis.

## ASM HEALTH

The ASM Health section covered studies on the pathogenicity of several viruses of concern, drug design, and potential therapeutic outcomes. The ASM Health category highlights innovative strategies for tackling infectious diseases, with particular emphasis on antibiotic resistance and host–pathogen interactions. This focus aligns with the overarching goal of many studies in the field: identifying novel therapeutic targets that can fuel drug discovery. It underscores how mechanistic insight can ultimately translate into meaningful advances in human health.

Dr. Nadine Alvarez (Center for Discovery and Innovation, Hackensack Meridian Health) presented her work on genetic and immunological profiling of recent SARS-CoV-2 omicron subvariants, providing insights into immune evasion and infectivity in monoinfections and co-infections. SARS-CoV-2 is responsible for the COVID-19 pandemic, with 778 million cases and 7 million deaths. Although the WHO declared an end to the global emergency in 2023, SARS-CoV-2 continues to persist and mutate. Current PANGO lineages of Omicron (e.g., JN.1, KP.3.1.1) harbor several novel spike protein mutations. Therefore, JN.1 and KP.3.1.1 subvariants in the recent isolates were selected for study. These recent omicron subvariants cannot be neutralized by commercially available antibodies, indicating an immune escape. SARS-CoV-2 has high incidences of co-circulation with influenza and RSV. The effect of co-circulation was studied in monoculture and co-culture of SARS-CoV-2 with other respiratory viruses using the *in vitro* air-liquid interface (ALI) model with human bronchial epithelial cells to replicate the lower respiratory tract environment. Co-culture with influenza-H1N1 and RSV resulted in higher infection and damage of bronchial airway epithelium, especially by 5–6 days. Viral quantification revealed that Omicron subvariants produced higher viral titers over time, indicating enhanced infectivity in lower airway cells—a surprising finding given prior reports suggesting Omicron preferentially infects upper respiratory tissue. There was increased production of pro-inflammatory cytokines (IL-6, TNF-α, interferon-β) and anti-inflammatory cytokines (IL-10) in both monoculture and co-culture, particularly in Omicron monoinfections and co-infections. New SARS-CoV-2 Omicron subvariants show enhanced infectivity in the human bronchial airway epithelium compared with the ancestral strains. Future studies will be focused on using organoids as a better representative model of the human lung environment. These findings underscore the need for ongoing genomic surveillance and vaccine updates tailored to new spike mutations and highlight concerns about synergistic respiratory infections during seasonal outbreaks.

Several posters were presented on the design and development of antivirals against viral pathogens, such as *Flavivirus*, the Omicron subvariant of SARS-CoV-2, and Mpox strains.

## ASM APPLIED AND ENVIRONMENTAL MICROBIOLOGY

The category for ASM Applied and Environmental Microbiology encompassed broad themes exploring varying talks from the microbiome within host organisms to agricultural ramifications, highlighting the diversity and potential of these discoveries.

Diemquynh Nguyen (Rutgers University-Newark) presented her work on characterizing the gut microbiome of *Myrmecocystus* (honeypot ants), which store nutrient-rich fluids in replete workers to survive the arid North American habitats. Across animal taxa, the gut microbiome is important for nutrient metabolism, detoxification, and host fitness. Honeypot ants represent an intriguing case because repletes act as living reservoirs, storing nectar in distended crops and regurgitating it through trophallaxis to nourish nestmates during nutrient scarcity. This raises key questions: How do repletes prevent stored nutrients from spoiling, and what is the role of resident gut microbes? Using 16S rRNA sequencing of crop and midgut tissues, she found that microbiome composition is species-specific and organ-compartmentalized, with Fructilactobacillus, a lactic acid bacterium found in fermented foods, highly abundant in *M. mendax* and *M. mexicanus*. Alpha diversity analyses confirmed that midguts harbor significantly more bacterial diversity than crops. Analysis of relative abundance and ANCOM-BC identified *Fructilactobacillus*, *Zymobacter*, and *Acetobacter* as crop-enriched, while *Vibrio* and *Pestinibacter* were midgut-enriched. Future studies will be focused on tracking microbial localization across the digestive system to better understand microbiome community dynamics. This research will further test whether disrupting these communities affects nutrient stability or colony health, determining whether these symbionts are active partners in the remarkable food-storage strategy of honeypot ants.

Lylla Almosd (Rutgers University-New Brunswick) presented her work on the characterization of cultivable bacteria, MRSA, and antibiotic resistance genes in bioaerosols and manure from pig and cow barns. Considering the growing threat of antibiotic resistance, her research aimed to investigate whether MRSA could be transmitted to humans via farm bioaerosols. Using active PTFE filters and passive REPS samplers, she detected MRSA in pig barns and manure from both pig and cow barns, while cow barn bioaerosols were overall negative. Pig-derived aerosols also showed higher 16S rRNA and sul1 gene abundance, although normalization showed there was actually similar relative abundance between both barn types. These findings show that bioaerosols and PM2.5 (fine particulate matter with a diameter of 2.5 μm or less) may facilitate microbial dispersal and act as potential vectors for antibiotic resistance transmission. Future work will expand gene assays, perform microbial community sequencing, and, as suggested during the discussion, attempt to isolate and genotype MRSA strains to determine whether they are of human or animal origin. Overall, her presentation added important preliminary evidence to the growing recognition that bioaerosols from agricultural sources represent a significant, yet often overlooked, pathway in the transmission of antibiotic resistance.

## CONCLUSIONS

The fall 2025 symposium of the Theobald Smith Society highlighted many important areas of investigation by researchers in New Jersey, particularly molecular mechanisms that regulate microbial adaptation in changing environments and stress, factors contributing to pathogenicity, and the development of potential therapeutic strategies. The research highlights the importance of growing interdisciplinarity required to gain substantial insights into the microbial landscape. For a more detailed account of the meeting proceedings, including all abstracts, visit https://www.njmicrobe.org/fall-2025-symposium. Video recordings are accessible at the YouTube channel for the Theobald Smith Society.
